# Benchmarking Large Language Models from Open and Closed Source Models to Apply Data Annotation for Free-Text Criteria in Healthcare

**DOI:** 10.3390/fi17040138

**Published:** 2025-03-24

**Authors:** Ali Nemati, Mohammad Assadi Shalmani, Qiang Lu, Jake Luo

**Affiliations:** 1 Health Informatics Department, Zilber College of Public Health, University of Wisconsin, Milwaukee, WI 53211, USA; 2 Beijing Key Laboratory of Petroleum Data Mining, China University of Petroleum, Beijing 102249, China; 3 Health Informatics & Administration Department, Zilber College of Public Health, University of Wisconsin, Milwaukee, WI 53211, USA

**Keywords:** large language models, healthcare data annotation, multi-criteria decision analysis, closed source and open source models, evaluation metrics, human and LLM evaluation, decision-making in healthcare

## Abstract

Large language models (LLMs) hold the potential to significantly enhance data annotation for free-text healthcare records. However, ensuring their accuracy and reliability is critical, especially in clinical research applications requiring the extraction of patient characteristics. This study introduces a novel evaluation framework based on Multi-Criteria Decision Analysis (MCDA) and the Order of Preference by Similarity to Ideal Solution (TOPSIS) technique, designed to benchmark LLMs on their annotation quality. The framework defines ten evaluation metrics across key criteria such as age, gender, BMI, disease presence, and blood markers (e.g., white blood count and platelets). Using this methodology, we assessed leading open source and commercial LLMs, achieving accuracy scores of 0.59, 1, 0.84, 0.56, and 0.92, respectively, for the specified criteria. Our work not only provides a rigorous framework for evaluating LLM capabilities in healthcare data annotation but also highlights their current performance limitations and strengths. By offering a comprehensive benchmarking approach, we aim to support responsible adoption and decision-making in healthcare applications.

## 1. Introduction

Large language models (LLMs) have emerged as powerful tools in natural language processing, driving innovation across various industries, including healthcare [1,2]. The rapid expansion of both open and closed source LLMs marks a transformative shift in how data are processed, interpreted, and utilized, ushering in a new era of computational intelligence [3,4]. LLMs have demonstrated significant potential in extracting patient information from free-text clinical records [5]. Clinical notes, discharge summaries, and medical reports—key components of medical documentation—contain free-text descriptions of patient characteristics that provide invaluable insights for clinical decision-making, research, and education. Accurate extraction of this information is critical for enhancing patient care, supporting clinical decisions, advancing research, and informing policy-making [6]. However, a fundamental question remains: can we rely on LLMs to extract meaningful insights from free-text medical reports?

This study is designed to bridge critical gaps in evaluating LLMs for healthcare applications, addressing two complementary objectives: first, to propose a novel evaluation framework specifically tailored for the healthcare domain, leveraging advanced multi-criteria decision-making techniques to provide rigorous and context-specific benchmarks; second, to systematically assess the performance of LLMs in extracting diverse patient attributes, such as age, gender, BMI, disease presence, and hematological parameters, from unstructured clinical records, thus shedding light on their practical utility and limitations in this sensitive field [7].

Multi-Criteria Decision Analysis (MCDA) is a structured approach used to evaluate and prioritize alternatives based on multiple conflicting criteria [8]. In healthcare, this is particularly relevant as decisions often involve trade-offs between accuracy, efficiency, and ethical considerations. MCDA provides a systematic framework to weigh these criteria, ensuring a balanced evaluation that aligns with the complex demands of healthcare applications [9].

The TOPSIS (Technique for Order of Preference by Similarity to Ideal Solution) method, a key component of MCDA, ranks alternatives by comparing their distance to an ideal solution [10]. This technique identifies the best option as the one closest to the positive ideal (most desirable) and furthest from the negative ideal (least desirable). TOPSIS is particularly effective in contexts where multiple quantitative metrics must be balanced, making it well suited for evaluating LLMs based on diverse performance criteria such as precision, recall, semantic coherence, and completeness [9].

Although extensive research has focused on benchmarking LLMs for general tasks such as named entity recognition [11], relation extraction [12], and question answering [12], these studies often lack a focus on real-world clinical applications. In particular, there remains a critical gap in frameworks specifically designed to evaluate the nuanced task of extracting patient-specific characteristics from free-text clinical records, a process that requires balancing precision, relevance, and context-awareness. This study aims to address this gap, introducing a novel framework for evaluating LLMs against diverse criteria reflective of healthcare-specific needs [13].

To ensure a robust and nuanced evaluation, this study employs a hybrid methodology that integrates automated processes with human oversight. Automated scripts, utilizing metrics such as precision, recall, and F1-score, provide an initial layer of evaluation, particularly suited for large-scale data processing [14]. However, recognizing the limitations of automated systems in capturing the full context of clinical text, human evaluators are incorporated to validate outputs where scripts alone prove insufficient. By combining the methodological rigor of MCDA with the ranking capabilities of TOPSIS, this study introduces a robust framework for systematically evaluating LLMs. These methods ensure that healthcare-specific benchmarks incorporate not only technical performance metrics but also broader considerations such as reliability and relevance.

By providing a rigorous benchmark for LLM performance, this study seeks to facilitate the responsible adoption of these models in healthcare settings. The findings have significant implications for clinical trial recruitment, where accurate extraction of patient characteristics is crucial, as well as for personalized medicine, which relies on precise annotation of individual attributes. Furthermore, the proposed framework offers a pathway for operational efficiency by automating routine tasks, enabling healthcare professionals to focus on higher-value activities. Ultimately, this research aims to support the broader integration of LLMs into healthcare, ensuring they are used effectively and ethically to improve patient outcomes.

## 2. Methodology

### 2.1. Dataset

The data source for this study is patient characteristic descriptions, commonly known as patient eligibility criteria, which are used for screening patients for clinical research [15]. These descriptions are an essential part of clinical trial protocols and provide detailed information about the target patient population, including demographic, clinical, and laboratory parameters [16].

The dataset used in this study was acquired from the Clinical Trials Transformation Initiative (CTTI) [17], a public–private partnership that aims to develop and drive the adoption of practices that will increase the quality and efficiency of clinical trials [18]. CTTI maintains a comprehensive database of clinical trial protocols, which includes patient eligibility criteria from a wide range of therapeutic areas and study designs.

**Figure 1.**
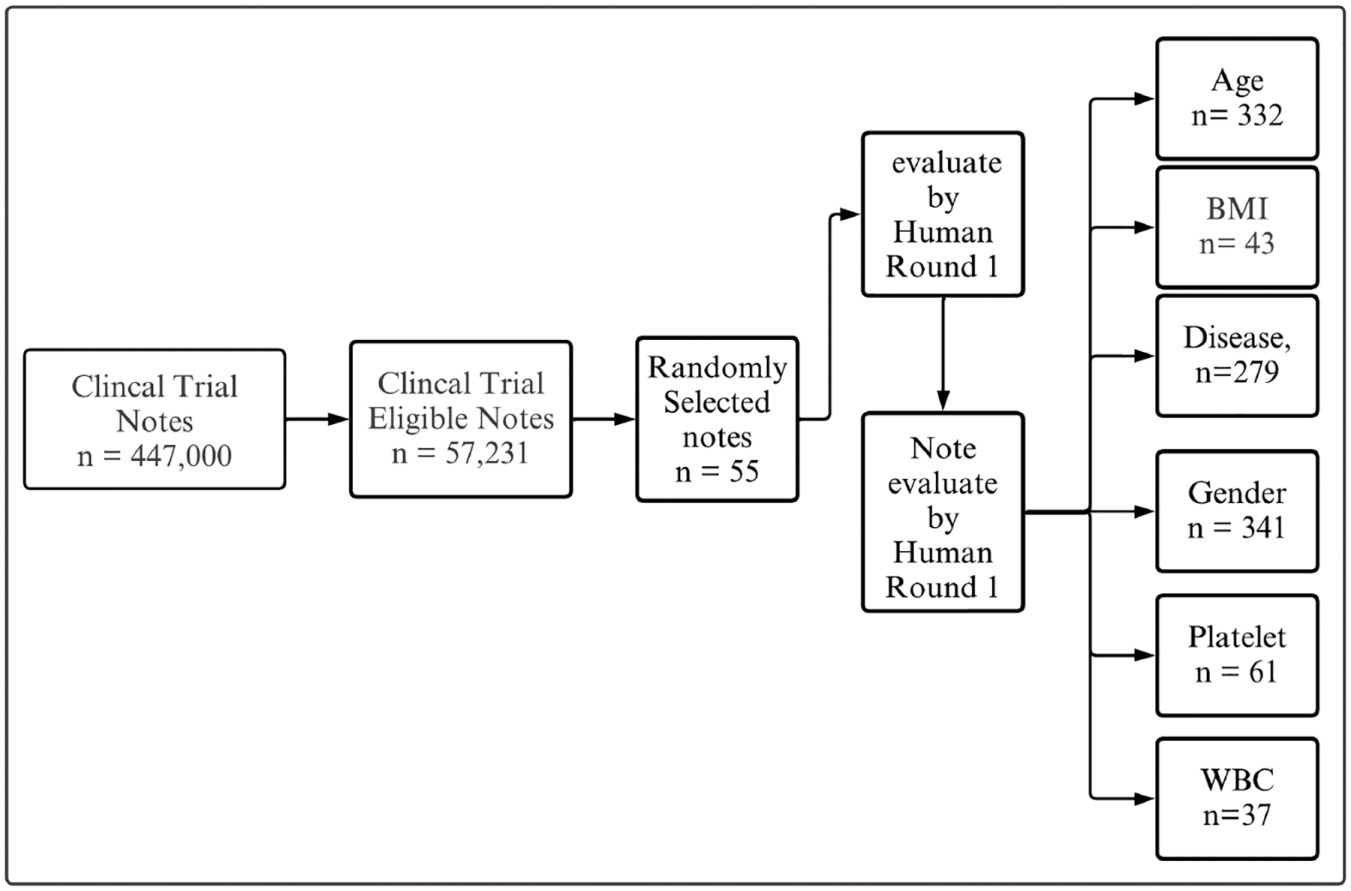
The process of evaluating clinical trial notes from an initial dataset, filtering eligible notes, and selecting 500 for human evaluation.

Figure 1 illustrates the process of evaluating clinical trial notes. Initially, a large dataset of clinical trial notes, totaling 447,000, is filtered down to identify eligible notes, resulting in a subset of 57,231 notes. From this eligible subset, 500 notes are randomly selected for further evaluation.

For our evaluation tests, we randomly selected a sample of patient eligibility criteria descriptions from the CTTI database. The sample size was determined based on computational power considerations and the practical feasibility of processing large datasets. Consequently, we narrowed the dataset to approximately 500 patient criteria descriptions for each of the evaluation tests, ensuring a diverse and representative subset. This approach balances the need for generalizability with the limitations of computational resources. These selected notes undergo two distinct rounds of human evaluation. In the first round, we meticulously review and annotate every piece of context, then cross-check and validate each text against specific categories for all criteria. This process is referred to as establishing the “ground truth”, as we are confident that the results are accurate and have been double-checked. Then, the notes are categorized based on specific criteria, including age, BMI, disease presence, gender, platelets count, and white blood cell (WBC) count. The counts for each category are as follows: Age (n = 332), BMI (n = 43), Disease (n = 279), Gender (n = 341), Platelets (n = 61), and WBC (n = 37). This systematic approach ensures a thorough and categorized evaluation of the clinical trial notes based on these critical parameters. By integrating the flowchart’s process with our evaluation methodology, we ensure that each clinical trial note is comprehensively evaluated and validated, providing a robust framework for patient eligibility criteria assessment. This process involves comparing the LLM’s output with ground truth answers provided by our team for specific criteria, including age, gender, BMI, platelets count, white blood cell (WBC) count, and disease categories. To generate the ground truth, we prompt GPT4 to provide initial results based on the specified categories. These results are then validated by human experts who carefully review and verify each extracted characteristic. To ensure the accuracy and reliability of the ground truth, a second round of validation is conducted, where the results are rechecked to confirm their correctness. This rigorous validation process ensures that the ground truth used for comparison is precise and trustworthy.

The patient eligibility criteria descriptions in our dataset cover a broad spectrum of medical conditions, including but not limited to cardiovascular diseases, oncology, neurological disorders, and infectious diseases. The descriptions also encompass various study types, such as randomized controlled trials, observational studies, and pragmatic trials, as contained in a registry and a database of publicly and privately supported clinical studies across the globe. This diversity in the dataset allows for a comprehensive evaluation of LLM performance across different clinical domains and research settings.

### 2.2. Data Preprocessing

To ensure dataset quality and consistency, we conducted data preprocessing steps, including text cleaning, anonymization, and standardization by removing stop words and applying a hybrid approach of stemming and lemmatization. These steps were crucial to remove any personally identifiable information, resolve inconsistencies in the data format, and prepare the dataset for input into the LLMs [19]. In our research, we categorize the dataset into two primary types: raw and clean data. Raw data refers to the dataset in its original form, without undergoing any preprocessing. On the other hand, clean data, as the term suggests, involves refining the text through various preprocessing steps to enhance its quality and consistency. These steps include removing stop words to reduce noise in the text, correcting encoding mismatches or corruptions (e.g., removing characters like “ï¿½?ï¿½”), and standardizing mathematical symbols for uniformity. For example, inconsistent representations such as “16>=” and “=<16” in different rows are unified to a single format, “=<16”. Furthermore, in addressing gender criteria, variations such as “women”, “a pregnant person”, or “Female” are standardized to “female” to maintain consistency across the dataset.

Our preprocessing methodology employs a context-sensitive hybrid approach of stemming and lemmatization to reduce token variability while ensuring linguistic consistency. Rather than applying these techniques uniformly, we strategically implemented them based on the nature of the text segments being processed. For sections containing medical terminology (e.g., rheumatoid arthritis, nephritis, thrombosis), lemmatization was preferred to preserve the precise meaning of these terms. Examples include the following: “Patients with thromboses” was transformed to “Patients with thrombosis” and “Diagnosed with nephropathies” was converted to “Diagnosed with nephropathy”. This ensures that words retain their medically relevant base form, preventing unintended distortions that could arise from stemming. Conversely, for common eligibility criteria or exclusion terms (e.g., “patients must”, “requirement of”), stemming was applied to standardize word forms where semantic precision was less critical. Examples include the following: “treated” reduced to “treat”, “evaluations” to “evalu”, and “conditions” to “condit”. This balanced approach leverages both the efficiency of stemming (reducing computational complexity and vocabulary size) and the accuracy of lemmatization (ensuring grammatically significant words or ambiguous root forms are converted into their correct dictionary forms). For instance, while stemming quickly reduces variability, lemmatization handles irregular forms more accurately. To ensure the correctness and consistency of our preprocessing steps, we manually reviewed the processed data against the original text as ground truth validation. This verification process confirmed that key medical terms remained accurate and unaltered in their intended context, stemming did not compromise readability or meaning in general descriptive criteria, and the processed text accurately reflected the original information while maintaining linguistic consistency. By reducing variability while respecting the contextual needs of different text segments, this hybrid method minimizes data sparsity and improves the quality of the preprocessed data for subsequent analysis.

However, recognizing the critical importance of clinical terminology accuracy, we implemented a human validation process to ensure that no essential medical terms were lost during preprocessing. Our validation methodology involved a comparison between raw and preprocessed (clean) data, where human evaluators meticulously reviewed and confirmed that critical medical terms, such as “platelets count”, “white blood cell count”, and other key patient attributes, remained intact. This careful verification process ensured that essential clinical information was preserved while benefiting from text normalization techniques. By using a large, diverse, and well-curated dataset from a reputable source like CTTI, our study aims to provide a robust and reliable evaluation of LLM performance in extracting patient characteristics from free-text eligibility criteria descriptions. The insights gained from this evaluation can inform the development and implementation of LLMs in clinical research and support the identification of eligible patients for clinical trials.

### 2.3. Designing Effective Prompts

**Figure 2.**
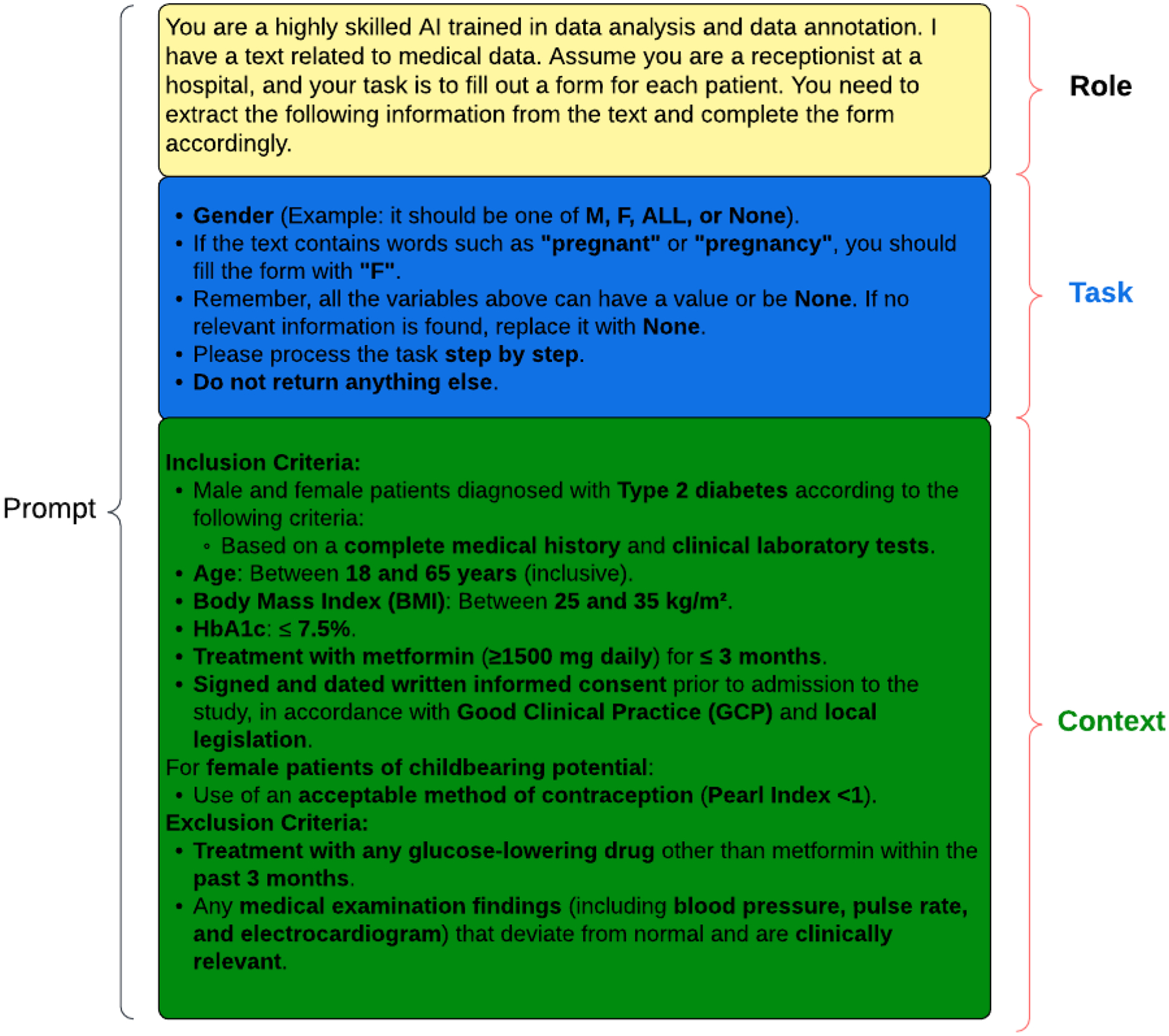
A structured framework for prompt construction in language model benchmarking, highlighting the integration of role, context, and task elements to enhance model performance evaluation.

Prompt design plays a crucial role in eliciting accurate and relevant responses from language models [20]. In this study, we propose a structured approach to prompt design, which we call “Designing Effective Prompts” (Figure 2). This methodology emphasizes the importance of three key elements: role, context, and task. While not all three elements are required in every prompt, their precise definition and thoughtful integration are essential for optimizing the performance of language models.

The role element defines the identity or function that the language model should assume in a given scenario. The language model could be prompted to act as a highly skilled data analyst and annotator, or as a hospital receptionist processing patient information. For example, consider the following:

You are an intelligent recipient for a doctor’s office. Extract the following patient-related information from the text: Extract disease. Look for the following diseases: hepatitis, HIV, cardiovascular disease, cancer, and diabetes. This information is being gathered to ensure accurate disease tracking and patient care management based on these specific criteria.

In our study, we are using these criteria to enhance the precision of disease identification and to streamline the processing of patient information. By specifying the role, we provide the language model with a clear understanding of the perspective it should adopt when responding to the prompt.

The task element provides the language model with the necessary background information and guidelines to perform the task effectively. In the task of patient eligibility criteria extraction, the task element might include instructions on how to interpret and annotate specific patient characteristics, such as gender, age, or medical conditions. This process can utilize the method of few-shot learning to enhance the model’s understanding and accuracy in performing the task [20].

For instance, the context element could specify the use of standardized markers like “M”, “F”, or “ALL” for gender, or provide guidance on assigning “F” when pregnancy is mentioned.

The context element clearly outlines the specific objectives or actions the language model is expected to accomplish. In our study, the context element focuses on identifying and extracting patient characteristics relevant to eligibility criteria for clinical trials. This could include identifying patients with specific medical conditions (e.g., type 2 diabetes), demographic characteristics (e.g., age, gender), or clinical parameters (e.g., body mass index, etc.).

By combining these three elements, “Designing Effective Prompts” offers a structured and adaptable approach to evaluate the capabilities of language models across various tasks and scenarios [21]. This methodology allows for the creation of targeted prompts that assess the language model’s ability to understand and extract patient characteristics from free-text eligibility criteria descriptions.

To ensure the effectiveness and reliability of our prompts, we conducted a thorough literature review and consulted with domain experts, including clinicians, researchers, and data annotators [22]. We also performed iterative testing and refinement of the prompts to optimize their clarity, specificity, and comprehensiveness.

### 2.4. LLM Data Extraction

To test the ability of large language models (LLMs) to extract patient characteristics from text, we passed prompts to various models asking them to identify specific criteria, such as age, gender, and other eligibility-related details, from a dataset. Each LLM was given a standardized prompt designed to extract relevant patient characteristics from free-text descriptions, which included eligibility criteria in clinical studies. Multiple LLMs, including GPT3.5, GPT4, Bard, Mistral, and Llama, were utilized to perform this task. The responses from these models were collected and saved for subsequent analysis. The evaluation involved using both raw and cleaned versions of the dataset, allowing us to assess the impact of data preprocessing on the models’ performance. This comparison provided insights into the robustness and adaptability of the LLMs in handling varying data quality. The extracted information was analyzed for accuracy, consistency, and completeness, focusing on specific metrics such as precision, recall, and F1-score. These findings highlighted the strengths and limitations of each model in processing free-text data and emphasized the importance of preprocessing in improving extraction accuracy and reliability. For all experiments, we set the temperature parameter to 0.0001, ensuring near-deterministic outputs. This choice minimizes randomness in model responses, making the results more stable across multiple runs. Additionally, to further control the generation process, we used the default top-k to 1 and top-p to 0 of each model, as they varied based on their underlying architectures and API constraints.

### 2.5. LLM Evaluation Design

In order to assess the performance of LLMs in extracting patient characteristics from free-text eligibility criteria descriptions, it is crucial to establish a comprehensive and multifaceted evaluation framework. The evaluation of LLMs in this context is essential for several reasons:

1. Ensuring accuracy and reliability: Extracting patient characteristics from free-text descriptions requires a high level of accuracy to support clinical decision-making and research. Evaluating LLMs helps ensure that the extracted information is reliable and can be trusted for downstream applications [23].
2. Assessing generalizability: LLMs should be able to perform well across a diverse range of eligibility criteria descriptions, covering various therapeutic areas, study designs, and patient populations. A comprehensive evaluation framework allows us to assess the generalizability of LLMs and identify potential limitations or biases [24].
3. Facilitating model selection and improvement: By evaluating multiple LLMs using a standardized framework, we can compare their performance, identify strengths and weaknesses, and select the most suitable models for specific tasks [23]. Moreover, the evaluation results can guide the development and fine-tuning of LLMs to enhance their performance in extracting patient characteristics [25,26].

To address these objectives, we propose an evaluation framework that incorporates multiple metrics, each capturing different aspects of LLM performance (Figure 2). The rationale for using multiple metrics is to provide a holistic assessment of LLMs, considering factors such as accuracy, relevance, consistency, and coherence [25]. By combining these metrics, we aim to obtain a more comprehensive understanding of LLM performance and make informed decisions regarding their deployment in clinical research settings.

The evaluation metrics included in our framework are as follows:

1. Human Validation: This metric involves comparing the LLMs’ outputs with ground truth answers provided by human experts for specific criteria such as BMI, age, gender, platelets count, and white blood cell count [27]. Human validation ensures that the LLMs’ performance is benchmarked against the gold standard of human judgment [27].
2. F1-Score, Precision, and Recall: These metrics, derived from the BERTScore methodology [28], assess the LLMs’ ability to generate outputs that match the ground truth data. By computing the similarity between tokens in the LLM output and the ground truth, we quantify each model’s accuracy and completeness in extracting relevant information.
3. Semantic Textual Similarity: Using the Sentence Transformer library with the “all-mpnet-base-v2” model, we measure the semantic similarity between the ground truth data and the LLM output. This metric captures the semantic proximity of sentences, providing insights into the model’s capacity to generate coherent and meaningful outputs [26,29], and is inspired by the DeepEval framework [30].
4. Factual Consistency: Factual consistency, a critical metric inspired by the DeepEval framework [30], was used to evaluate the alignment of the LLMs’ outputs with the input data. This metric is particularly important in the clinical domain, where inaccuracies could have serious consequences for patient care and research integrity [26]. In our study, factual consistency was assessed by employing string-matching algorithms. These algorithms compared key terms and phrases extracted by the LLMs, such as age, gender, and other eligibility-related criteria, against their counterparts in the input data. This approach ensured that the extracted terms matched the intended meanings in the clinical context, reducing the risk of misinterpretation or error.
  The choice of string-matching algorithms was informed by their ability to handle synonyms, abbreviations, and variations in phrasing common in free-text clinical descriptions. For instance, terms like “male” and “M” were mapped consistently, and numerical data such as age ranges were validated against explicit and implied descriptions in the input text. This level of granularity provided a robust mechanism to ensure that the outputs generated by the LLMs were factually aligned with the source material.
  In addition to factual consistency, qualitative metrics such as coherence, consistency, fluency, and relevance were evaluated. These metrics, inspired by the Unified Multi-dimensional Evaluator framework [26,31,32], provided a nuanced understanding of the LLMs’ language generation capabilities. Coherence assessed the logical flow and structure of the outputs, ensuring that the extracted information formed a comprehensible narrative. Consistency evaluated whether the outputs remained uniform across different sections of the generated text, avoiding contradictions or deviations. Fluency measured the linguistic quality of the text, focusing on grammar, syntax, and readability, while relevance ensured that the generated outputs were directly aligned with the input prompts and data.
  These qualitative metrics were applied systematically to compare the performance of multiple LLMs. The evaluation revealed key insights into the models’ strengths and limitations, such as their ability to handle complex eligibility criteria with overlapping or ambiguous terms. By combining the quantitative assessment of factual consistency with these qualitative measures, our study offered a comprehensive framework for evaluating the performance of LLMs in extracting reliable and contextually appropriate outputs from free-text clinical data.

To integrate these multiple metrics and identify the top-ranking LLMs, we employ Multi-Criteria Decision Analysis (MCDA) using the Technique for Order of Preference by Similarity to Ideal Solution (TOPSIS) [33]. TOPSIS allows us to consider the relative importance of each metric and rank the LLMs based on their proximity to the ideal solution [34]. By applying MCDA, we can make informed decisions about the most suitable LLMs for extracting patient characteristics from free-text eligibility criteria descriptions.

**Figure 3.**
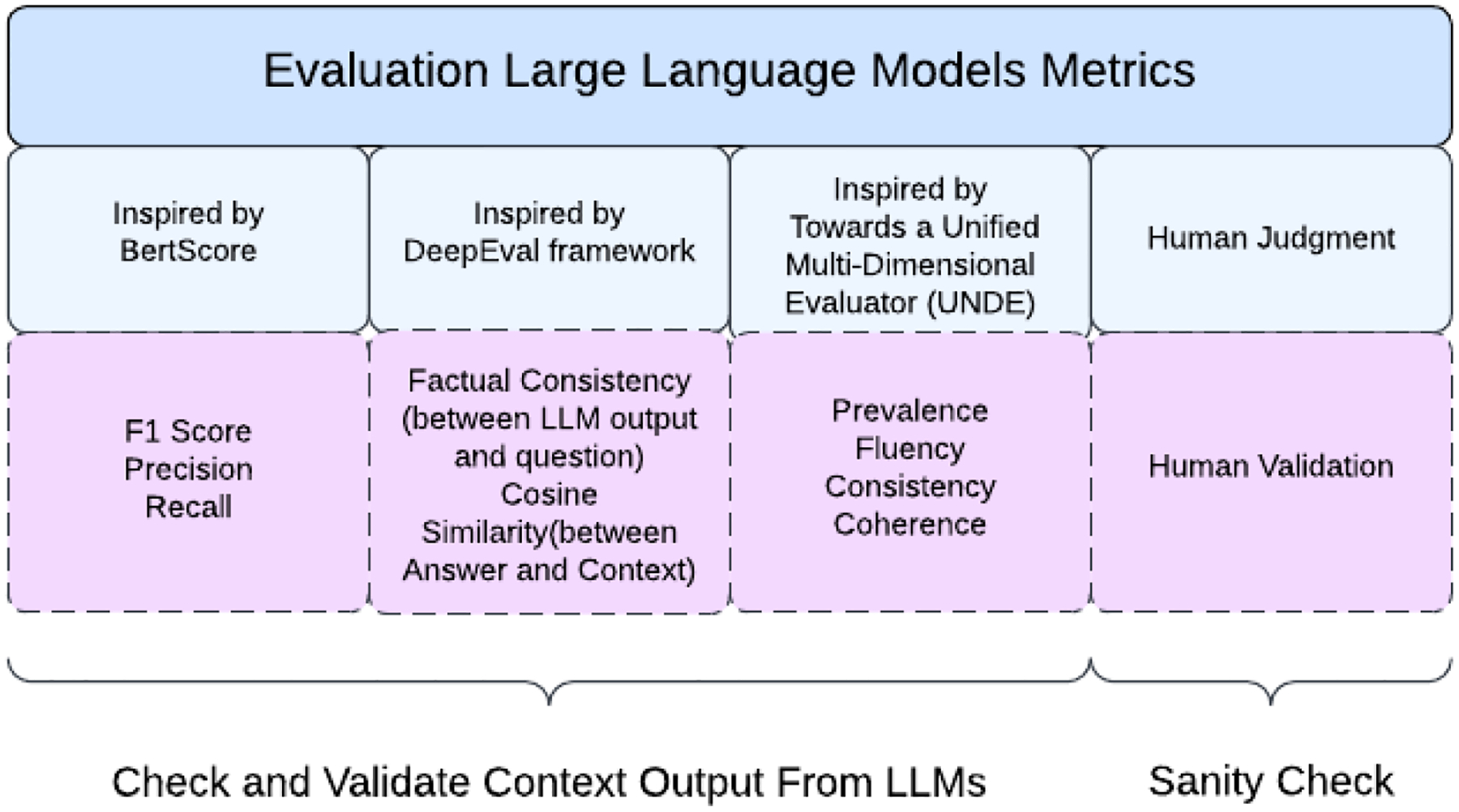
Evaluation metrics for large language models.

Figure 3 presents a multi-dimensional evaluation framework for large language models, integrating automated metrics (inspired by BertScore, DeepEval, and UNDE) with human judgment. These measures encompass precision-based metrics (F1, precision, recall), factual consistency, semantic similarity, and coherence to ensure a comprehensive assessment of model outputs.

### 2.6. Human Validation

To assess the performance of LLMs in extracting patient characteristics from free-text eligibility criteria descriptions, we incorporate a human validation process. Human validation is critical for ensuring the accuracy and reliability of automated information extraction from medical texts [35]. Human evaluators were provided with comprehensive and detailed annotation guidelines to ensure consistency and accuracy in the evaluation process. Annotation guidelines included a standardized set of instructions outlining the criteria for labeling each patient characteristic, including definitions and examples for age, gender, BMI, platelets count, WBC count, and disease categories. To ensure inter-rater reliability, each clinical trial note was independently evaluated by two annotators.

#### 2.6.1. Scoring System

For age, gender, BMI, platelets count, and WBC count, we employ a binary scoring system:

1. **Score of 1**: Assigned if the LLM’s output accurately extracts the information for the specified criterion.
2. **Score of 0**: Assigned if the LLM’s output fails to mention or incorrectly mentions the information for the specified criterion.

This binary scoring system allows us to evaluate the LLM’s accuracy in extracting these key patient characteristics.

For evaluating the LLM’s performance in identifying disease-related information, we consider five main disease categories: cancer, hepatitis, HIV, diabetes, and cardiovascular diseases. We use a proportional scoring system based on the number of correctly identified disease categories:

1. **Score of 0.2**: Assigned for each correctly identified disease category.
2. **Score of 0.4**: Assigned for two correctly identified disease categories.
3. **Score of 0.6**: Assigned for three correctly identified disease categories.
4. **Score of 0.8**: Assigned for four correctly identified disease categories.
5. **Maximum score of 1**: Achieved if the LLM’s output accurately includes all five disease categories.

The proportional scoring system acknowledges the LLM’s ability to identify multiple disease categories and provides a more granular assessment of its performance.

#### 2.6.2. Overall Human Validation Score

To calculate the overall human validation score for each LLM, we first compute the average score for age, gender, BMI, platelets count, and WBC count:

(1) $$\text{Avg Score(Age, Gender, BMI, Platelets, WBC)}=\frac{\sum_{i=1}^{n}\text{Scor}{\text{e}}_{\text{i}}}{n}$$

where *n* is the total number of criteria (i.e., five) and Score_*i*_ is the binary score for each criterion.

Next, we calculate the average score for the disease categories:

(2) $$\text{Avg Score (Disease Categories)}=\frac{\sum_{j=1}^{m}{\text{Score}}_{j}}{m}$$

where *m* is the total number of disease categories (i.e., five) and Score_*j*_ is the proportional score for each category.

Finally, we compute the overall human validation score by taking the average of the two scores:

(3) $$\text{Human Validation Score}=\frac{1}{2}(\text{Avg Score(Age, Gender, BMI,Platelets, WBC)}+\text{Avg Score (Disease Categories)})$$

The human validation score ranges from 0 to 1, with higher scores indicating better performance in extracting patient characteristics from free-text eligibility criteria descriptions.

By using this clear and structured scoring system, we aim to provide a thorough and systematic assessment of LLM performance in the context of patient characteristic extraction. The human validation score serves as a crucial component of our evaluation framework, enabling the comparison of different LLMs and guiding their selection for clinical research applications.

### 2.7. F1-Score, Precision and Recall

To evaluate the performance of LLMs in extracting patient characteristics, we employ the BERTScore methodology. BERTScore provides a comprehensive evaluation by comparing the tokens of the ground truth data (*x*) with the LLM’s output $\left(\overset{ˆ}{x}\right)$. This comparison is performed using a pre-trained BERT model, which generates contextualized embeddings for each token [28].

The BERTScore calculation involves a greedy matching process to maximize the similarity score between the tokens of the ground truth and the LLM’s output. For each token in the ground truth, the most similar token in the LLM’s output is identified, and vice versa. This matching process allows for the computation of precision and recall scores.

Recall (*R*_*BERT*_) measures the proportion of tokens in the ground truth that are correctly captured by the LLM’s output. It is calculated as follows:

(4) $$R_{\text{BERT}}=\frac{1}{\left|x\right|}\sum_{x_{i}\in x}\underset{{\overset{ˆ}{x}}_{j}\in \overset{ˆ}{x}}{\text{max}}\;x_{i}^{⊤}{\overset{ˆ}{x}}_{j}$$

where *x*_*i*_ represents a token in the ground truth, ${\overset{ˆ}{x}}_{j}$ represents a token in the LLM’s output, and $x_{i}^{⊤}{\overset{ˆ}{x}}_{j}$ denotes the STS between their respective embeddings.

Precision (*P*_*BERT*_) measures the proportion of tokens in the LLM’s output that are relevant to the ground truth [36]. It is calculated as follows:

(5) $$P_{\text{BERT}}=\frac{1}{\left|\overset{ˆ}{x}\right|}\sum_{\overset{ˆ}{x}j\in \overset{ˆ}{x}}\text{max}\;x_{i}\in xx_{i}^{⊤}{\overset{ˆ}{x}}_{j}$$

The F1-score (*F*_*BERT*_) is the harmonic mean of precision and recall, providing a balanced measure of the LLM’s performance [36]. It is calculated as follows:

(6) $$F_{\text{BERT}}=2\cdot \frac{P_{\text{BERT}}\cdot R_{\text{BERT}}}{P_{\text{BERT}}+R_{\text{BERT}}}$$

In our study, we calculate the F1-score, precision, and recall by applying the BERTScore methodology to the LLM’s output and the corresponding ground truth data. Instead of using the original BERT model, we employ the T5 model [37], which has been specifically designed for text-to-text tasks and has shown superior performance in various natural language processing applications [28].

### 2.8. Semantic Textual Similarity

To further evaluate the Semantic Textual Similarity (STS) between the ground truth data and the LLMs’ outputs, we employ the Sentence Transformer library. Sentence transformers are a set of pre-trained models that generate dense vector representations of sentences, capturing their semantic meaning [28]. These models have been shown to outperform traditional word embedding methods in various natural language processing tasks, including semantic textual similarity [28].

In our study, we utilize the “all-mpnet-base-v2” model, a state-of-the-art transformer model pre-trained on a large corpus of text data. This model has demonstrated excellent performance in encoding sentences into semantically meaningful vectors [38]. By leveraging the “all-mpnet-base-v2” model, we aim to capture the semantic proximity between the ground truth and each LLM’s output effectively [39].

The process of calculating STS using Sentence Transformers involves the following steps [39]:

Encoding the ground truth and LLM output: The “all-mpnet-base-v2” model is used to generate dense vector representations for each sentence in the ground truth and the corresponding LLM output. These vectors are obtained by passing the sentences through the pre-trained model, which learns to map semantically similar sentences to nearby points in the vector space. Once the vector representations are obtained, we calculate the STS between the ground truth and LLM output vectors. STS measures the cosine of the angle between two vectors in a multi-dimensional space. It ranges from −1 to 1, with higher values indicating greater semantic similarity. The semantic textual similarity between two vectors **a** and **b** is calculated as follows:

(7) $$\text{semantic textual similarity}=\frac{a\cdot b}{\left|a||b\right|}$$

where **a** · **b** denotes the dot product of the vectors, and |**a**| and |**b**| represent their Euclidean norms.

**Figure 4.**
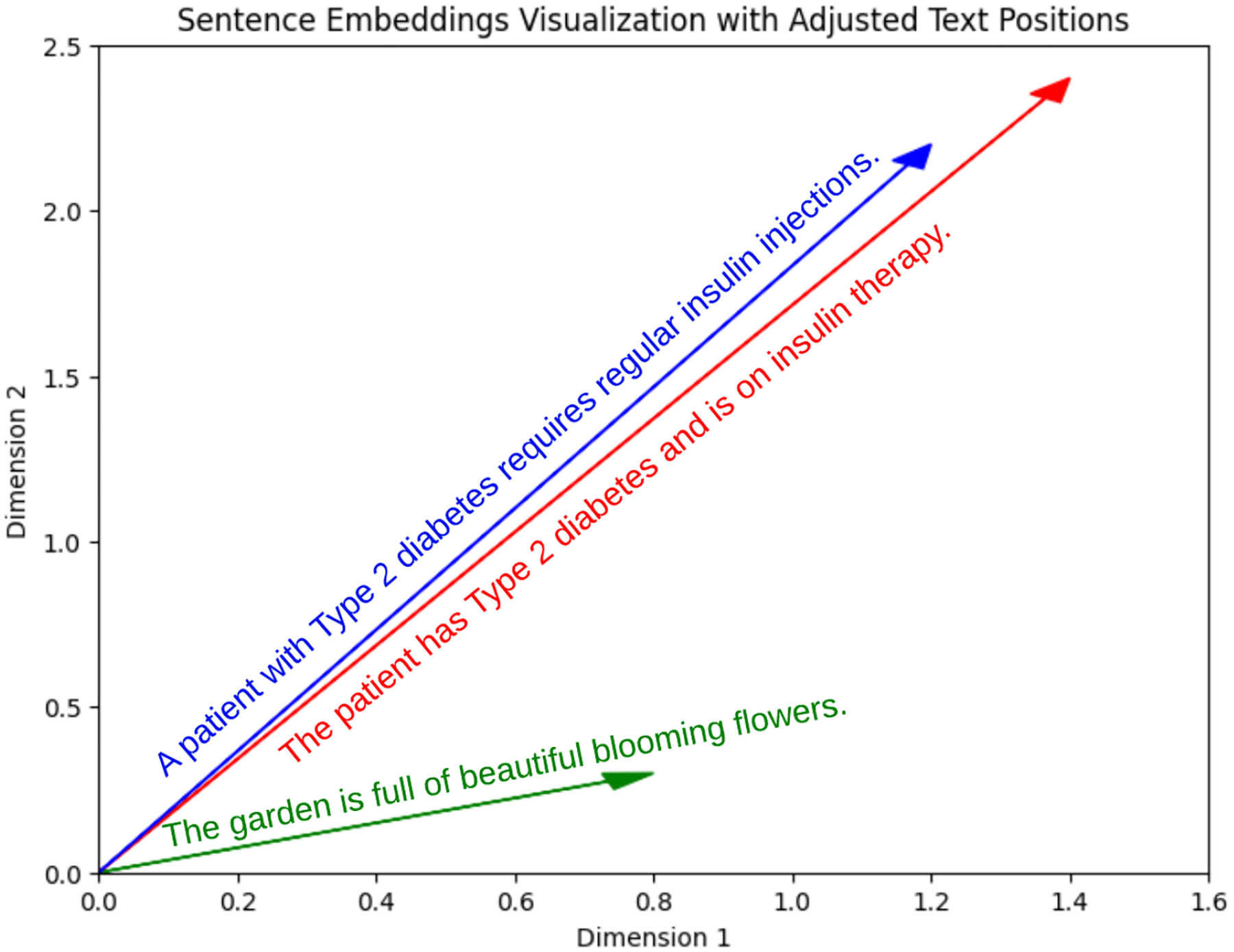
Semantic Textual Similarity.

Figure 4 demonstrates how sentences with similar semantic content are positioned closer together in the embedding space, while unrelated sentences appear farther apart.

As an illustrative example (Figure 4) using sentence embeddings from the “all-MiniLM-L6-v2” model, we explored semantic relationships centered around the sentence “The new movie is awesome for learning a new language”. This analysis revealed clear patterns: notably, the sentence exhibited a robust similarity (similarity score: 0.9299) with “The new movie is so exciting to learn a new language”, indicating a strong thematic alignment. In contrast, sentences like “A man walks down the street” (similarity score: −0.0948) and “People are shopping in the mall” (similarity score: −0.0188) showed lower or negative similarities, highlighting distinct semantic contrasts. This example showcases the model’s ability to discern and quantify semantic nuances effectively across different contexts.

In our framework, we used semantic similarity across all categories. The semantic similarity scores generated offer a quantitative assessment of how closely the LLM’s output aligns with the ground truth. Scores near 1 denote a high level of semantic similarity, indicating that the LLM has successfully captured the meaning and context of the ground truth [29]. Conversely, lower scores suggest a semantic disparity between the two texts, indicating a greater divergence in meaning and context between the ground truth and the LLM’s output [29].

### 2.9. Factual Consistency

Factual consistency is a crucial aspect of evaluating the performance of LLMs in extracting patient characteristics from free-text eligibility criteria descriptions [40]. Factual consistency measures the extent to which the information extracted by the LLM aligns with the factual details present in the ground truth data. This evaluation determines how factually correct an LLM application is based on the respective context as given by the evaluation dataset.

To assess factual consistency, we use automated methods that compare extracted entities indicating disease, age, gender, BMI, platelets count, and WBC count between the LLM’s output and the ground truth. This comparison can be performed using string matching algorithms or more advanced techniques like named entity recognition and normalization.

The factual consistency score is calculated as the proportion of correctly extracted factual details out of the total number of relevant facts present in the ground truth [41]. A higher factual consistency score indicates that the LLM is able to accurately extract and represent the key information from the eligibility criteria descriptions [42].

### 2.10. Coherence, Consistency, Fluency, and Relevance

In addition to the quantitative metrics discussed earlier, we also evaluate the qualitative aspects of the analyzed LLMs’ (GPT3.5, GPT4, Bard, Llama2, Mistral) output, including coherence, consistency, fluency, and relevance [31]. These factors are essential for ensuring that the extracted patient characteristics are presented in a clear, understandable, and contextually appropriate manner [31].

Coherence refers to the logical flow and structure of the LLM’s output. It assesses whether the extracted information is organized in a coherent and meaningful way, making it easy for readers to comprehend [31,43,44].

Consistency measures the uniformity of the extracted information across different parts of the LLM’s output [31]. It ensures that the patient characteristics are represented consistently throughout the generated text, without contradictions or discrepancies [44].

Fluency evaluates the linguistic quality of the LLM’s output. It assesses whether the generated text follows the grammatical and syntactic rules of the language, resulting in smooth and natural-sounding sentences [45].

Relevance gauges the extent to which the extracted patient characteristics are pertinent to the specific eligibility criteria and the overall context of the clinical study. It ensures that the LLM captures the most important and relevant information from the free-text descriptions.

To evaluate these qualitative aspects, we employ a combination of human evaluation and automated metrics. Human experts assess the coherence, consistency, fluency, and relevance of the LLM’s output using predefined rubrics or rating scales. Automated methods, such as language models trained on coherence and fluency datasets, can provide complementary scores.

### 2.11. Multi-Criteria Decision Analysis to Select Top Rank

To determine the top-performing LLM for extracting patient characteristics from free-text eligibility criteria descriptions, we employ Multi-Criteria Decision Analysis (MCDA) using the Technique for Order of Preference by Similarity to Ideal Solution (TOPSIS) [33].

TOPSIS is a well-established Multi-Criteria Decision Analysis (MCDA) method that systematically evaluates alternatives based on multiple criteria [46,47]. In our study, the alternatives are different large language models (LLMs) being evaluated, and the criteria include various evaluation metrics such as human validation scores, F1-scores, precision, recall, semantic textual similarity, factual consistency, coherence, consistency, fluency, and relevance. TOPSIS was chosen because it efficiently determines the relative performance of alternatives by measuring their closeness to an ideal solution. It provides a clear, objective, and mathematically grounded ranking of LLMs.

The TOPSIS methodology involves the following steps [48,49]:

1. **Constructing a decision matrix**: The performance scores of each LLM across all evaluation metrics are organized into a decision matrix. This matrix serves as the foundation for the comparison of alternatives.
2. **Normalizing the decision matrix**: The scores are normalized to ensure comparability across criteria with different scales. This step avoids the dominance of any particular metric due to its range or magnitude.
3. **Assigning weights to the criteria**: Each evaluation metric is assigned a weight based on its relative importance in assessing LLM performance. This ensures that critical metrics, such as human validation and F1-score, have greater influence.
4. **Identifying the Positive Ideal Solution (PIS) and Negative Ideal Solution (NIS)**: The PIS represents the best possible scores across all criteria, while the NIS represents the worst scores. These benchmarks serve as reference points for evaluating the alternatives.
5. **Calculating the Euclidean distance**: The distance of each LLM from the PIS ($D_{i}^{+}$ and NIS ($D_{i}^{-}$) is calculated using the Euclidean distance formula. This provides a robust measure of each model’s closeness to the ideal and worst cases.
6. **Computing the Relative Closeness to the Ideal Solution (RCIS)**: The RCIS for each LLM is computed using the formula
(8) $${\text{RCIS}}_{i}=\frac{D_{i}^{-}}{D_{i}^{+}+D_{i}^{-}}$$
  where $D_{i}^{+}$ is the distance of the *i*th LLM from the PIS, and $D_{i}^{-}$ is the distance from the NIS. Higher RCIS values indicate better-performing LLMs.
7. **Ranking the LLMs**: The LLMs are ranked based on their RCIS values, with the highest value indicating the best-performing model.

By applying the TOPSIS methodology, we achieve a comprehensive evaluation of the LLMs, considering multiple criteria and their relative importance [47]. TOPSIS ensures a transparent and objective ranking process, enabling the identification of the top-performing LLM that excels across various dimensions. This approach provides a reliable solution for extracting patient characteristics from free-text eligibility criteria descriptions [50].

### 2.12. Evaluate Framework Using MCDA

In this framework, we perform Multi-Criteria Decision Analysis (MCDA) to comprehensively evaluate the performance of LLMs using metrics such as human validation, precision, recall, semantic textual similarity, factual consistency, coherence, consistency, fluency, and relevance. The primary objective is to analyze and rank the LLM responses using specified weights and objectives, facilitating informed decision-making.

**Human Validation and Gold Standard Responses (A)**: Human validation holds the highest weight, as it ensures the accuracy and reliability of results. This process involves human annotation and comparison with gold standard answers within the context.

**Precision, Recall, and F1-Score (B)**: These core metrics evaluate the effectiveness of the LLM responses. They are equally weighted, emphasizing their critical role in determining accuracy and alignment with the gold standard answers.

**Semantic Textual Similarity and Factual Consistency (C)**: These metrics assess contextual relevance and truthfulness. They are assigned slightly lower weights than direct performance metrics but remain essential for validating contextual alignment and factual accuracy.

**Coherence, Consistency, Fluency, and Relevance (D)**: These qualitative metrics focus on the narrative quality and contextual appropriateness of the responses. They are assigned the lowest weights, ensuring balance in evaluating overall response quality.

The criteria weights are defined as [1, 1, 1, 1, 0.9, 0.9, 0.85, 0.85, 0.85, 0.85], with all metrics set to be maximized. The highest weights (1) are assigned to “Human Validation”, “F1-Score”, “Precision”, and “Recall”, reflecting their impact on accuracy. Slightly lower weights are assigned to semantic textual similarity and factual consistency, while qualitative metrics receive the lowest weights.

The Euclidean distance metric is used in TOPSIS because it effectively measures the straight-line distance in multi-dimensional space, ensuring a robust comparison of alternatives. Results are rounded to two decimal places for clarity. The function “perform_MCDA” processes the given DataFrame, applies the weights and objectives, and generates a comprehensive ranking of the LLMs.

**Figure 5.**
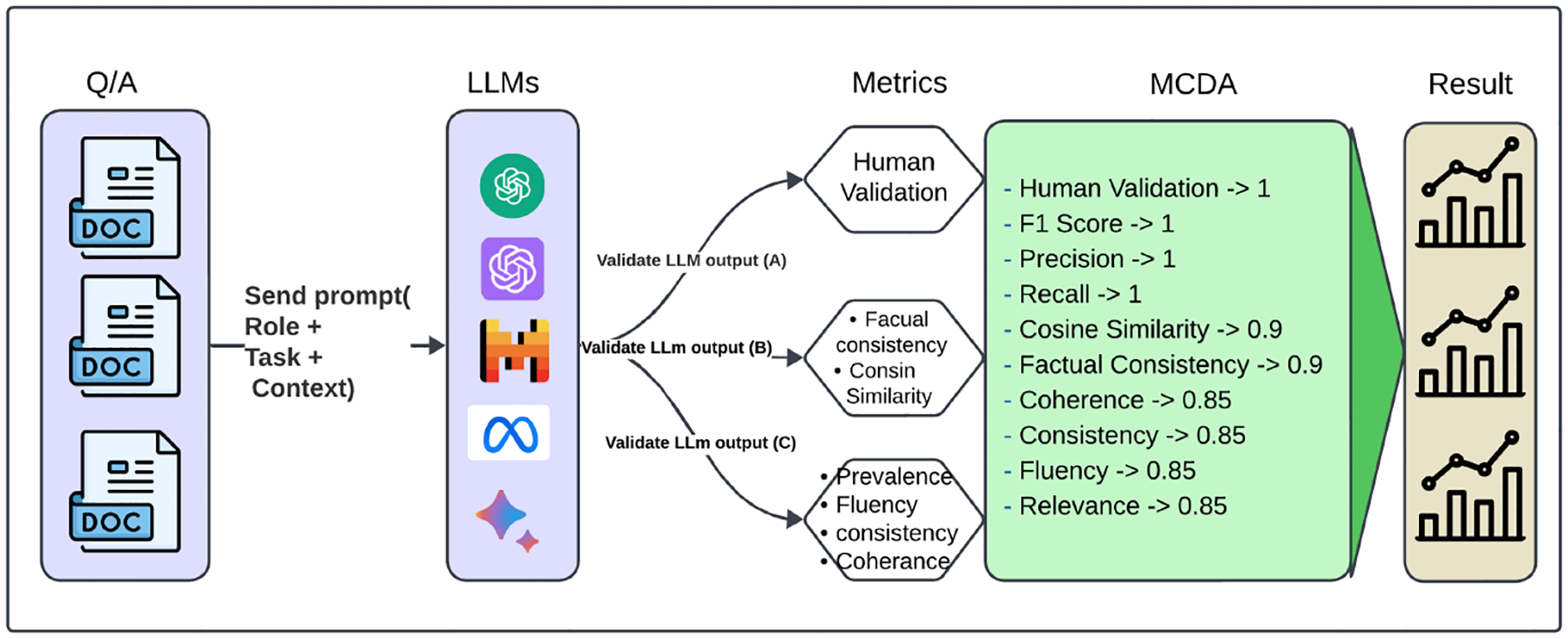
Evaluation of large language models metrics by applying MCDA. The figure illustrates the MCDA process, including the criteria weights, objectives, and resultant rankings.

In Figure 5, the evaluation is divided into three categories:

- **A (Gold Standard Answer + LLM Response + Context)**: Combines all elements for comprehensive validation and consistency.
- **B (Gold Standard Answer + LLM Response)**: Directly compares the gold standard answers with LLM responses to assess accuracy.
- **C (Context + LLM Response)**: Evaluates contextual suitability without considering the gold standard answer.

This systematic MCDA approach provides a transparent and reliable evaluation of the LLMs, identifying the most suitable model for extracting patient characteristics from free-text eligibility criteria descriptions. Building upon this methodology, the subsequent section presents the evaluation results, offering a detailed analysis of model performance across multiple health-related categories.

## 3. Results

**Figure 6.**
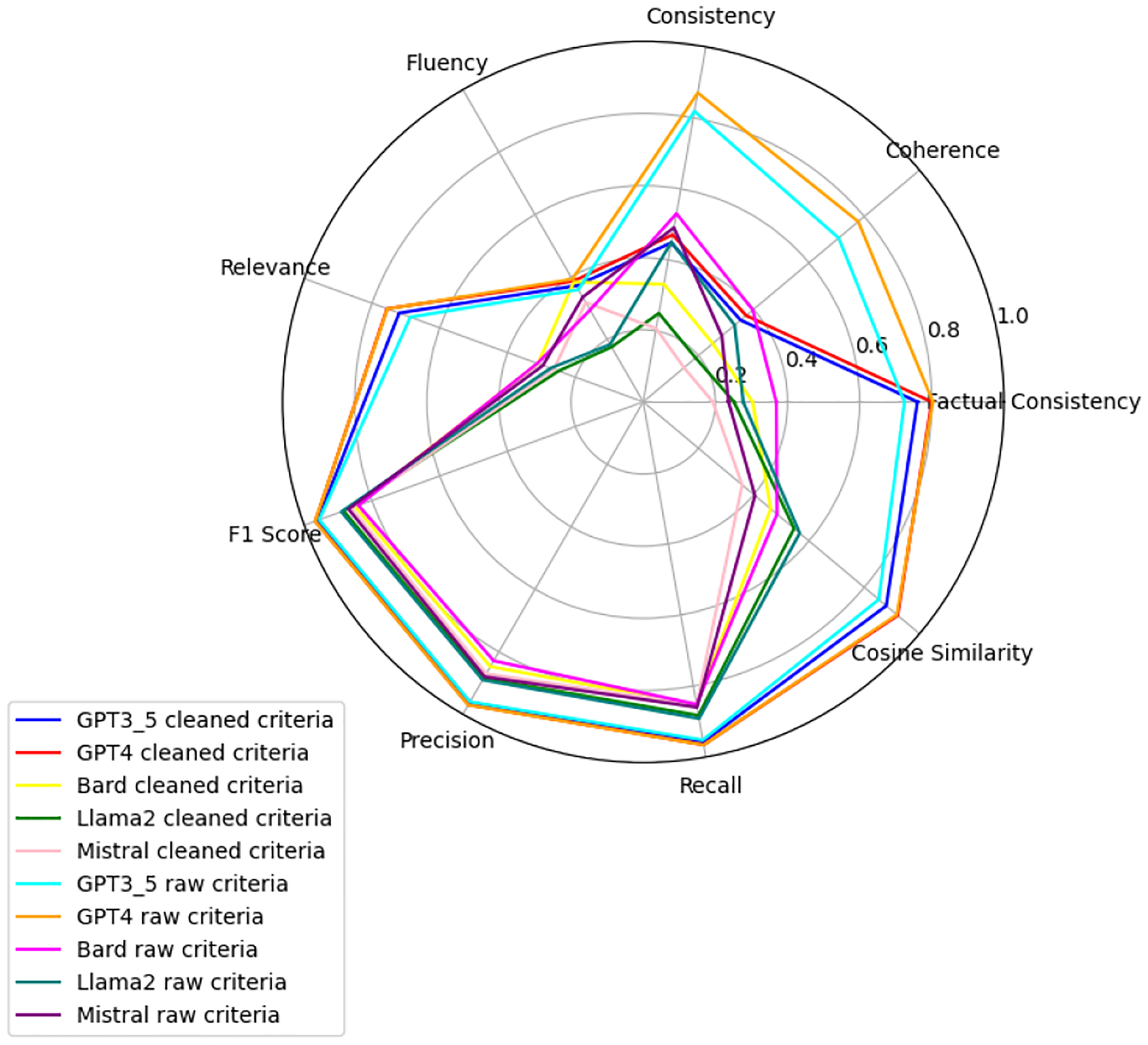
Age: all models.

**Figure 7.**
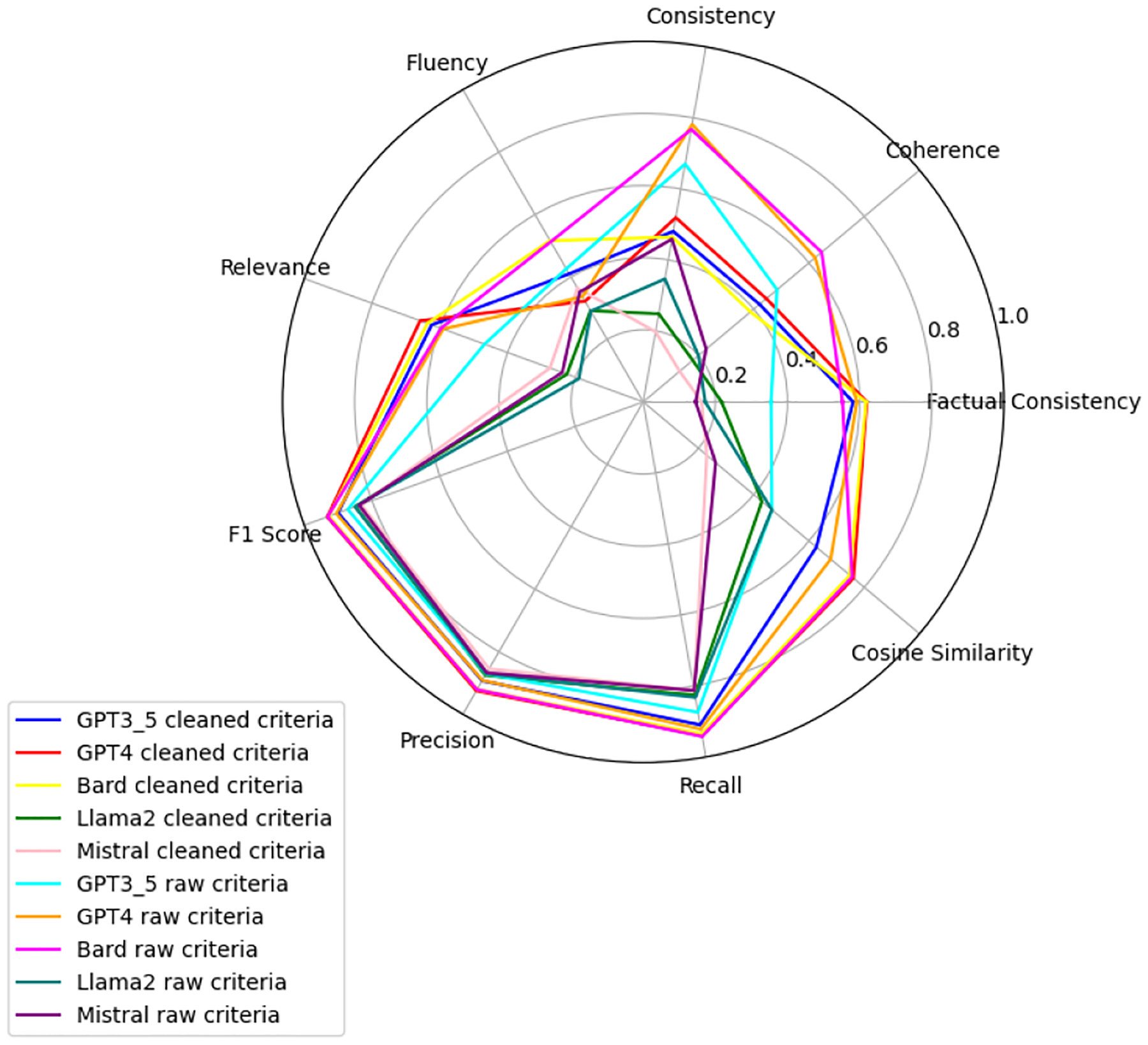
BMI: all models.

**Figure 8.**
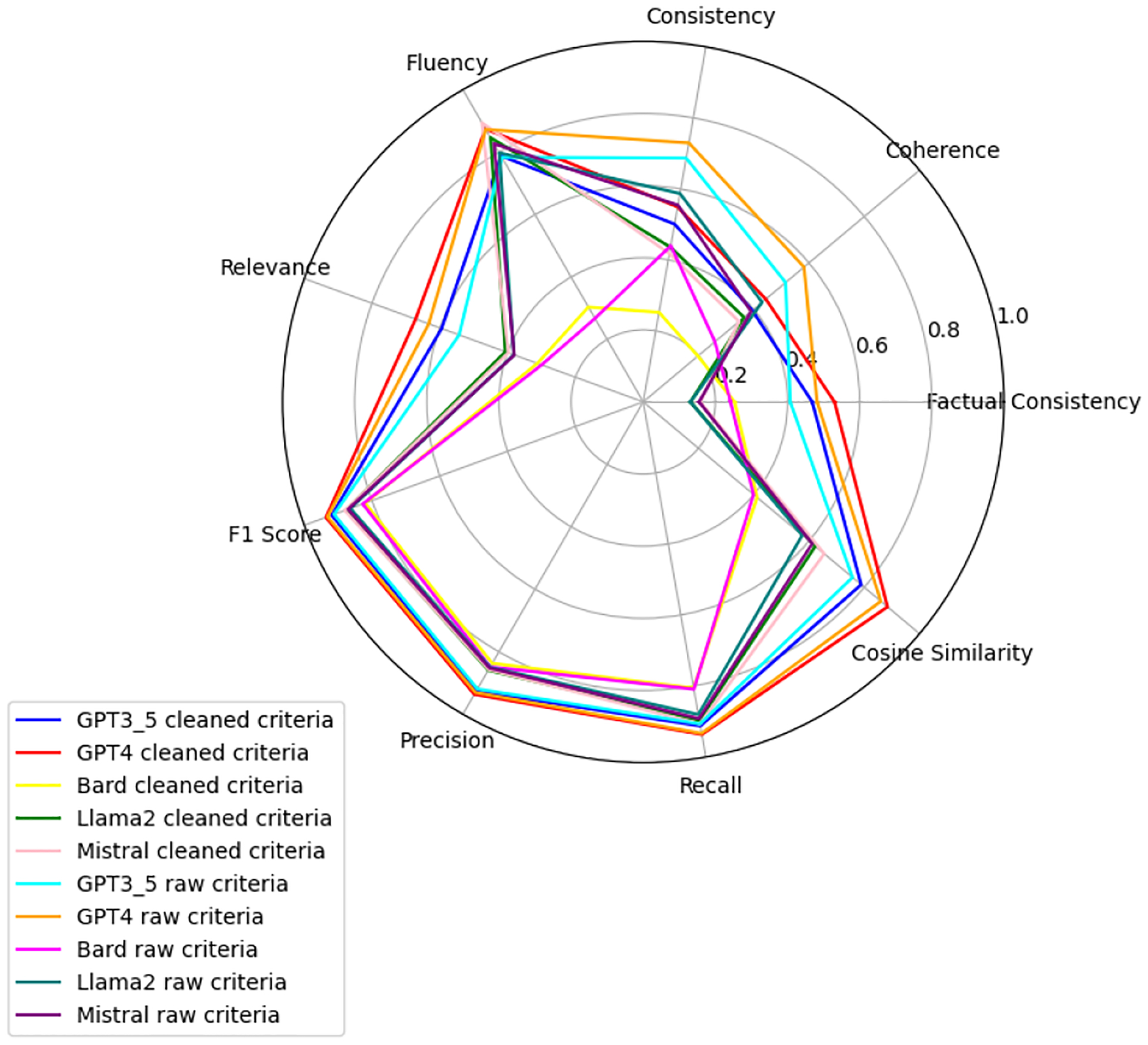
Disease: all models.

**Figure 9.**
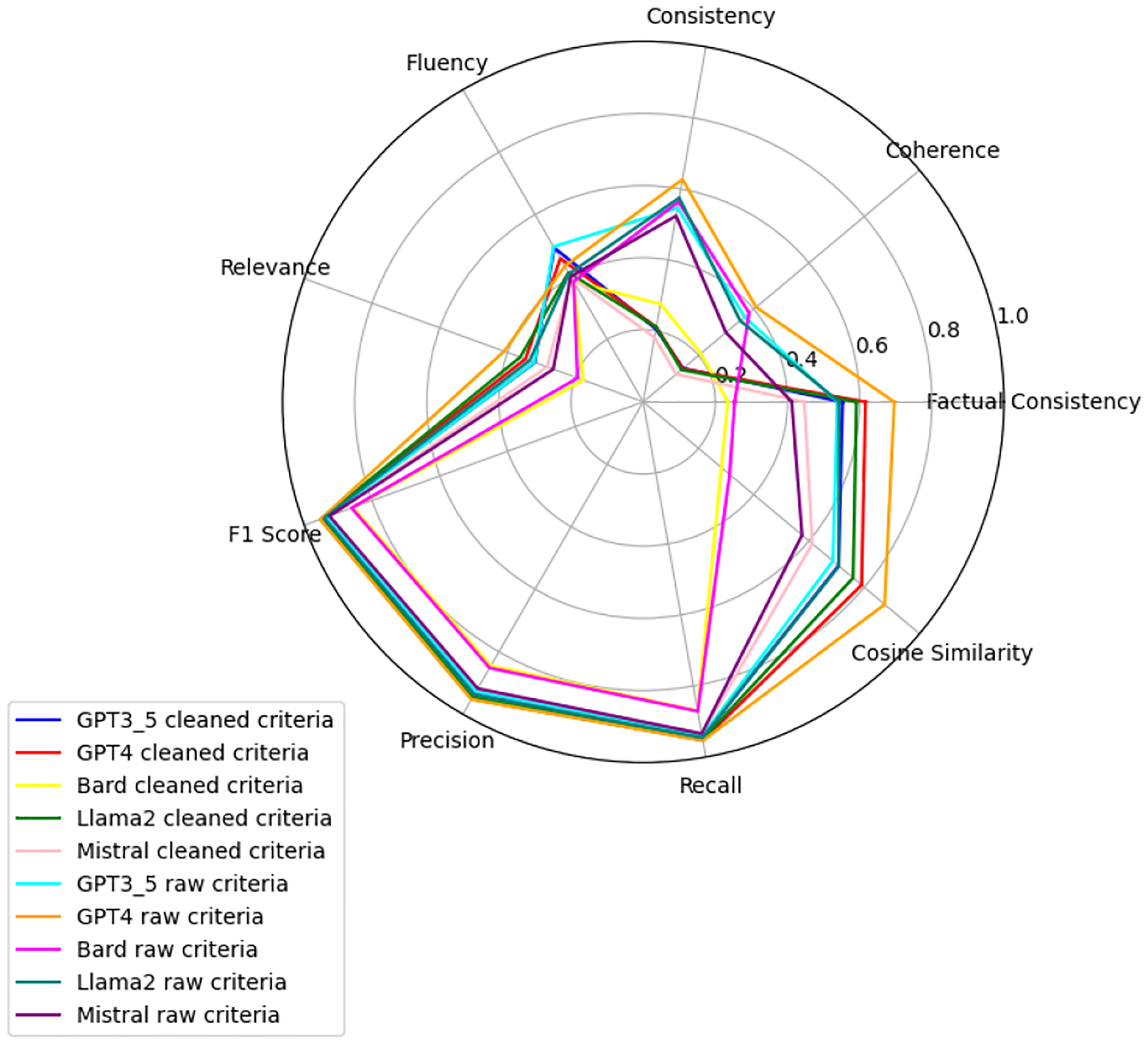
Gender: all models.

**Figure 10.**
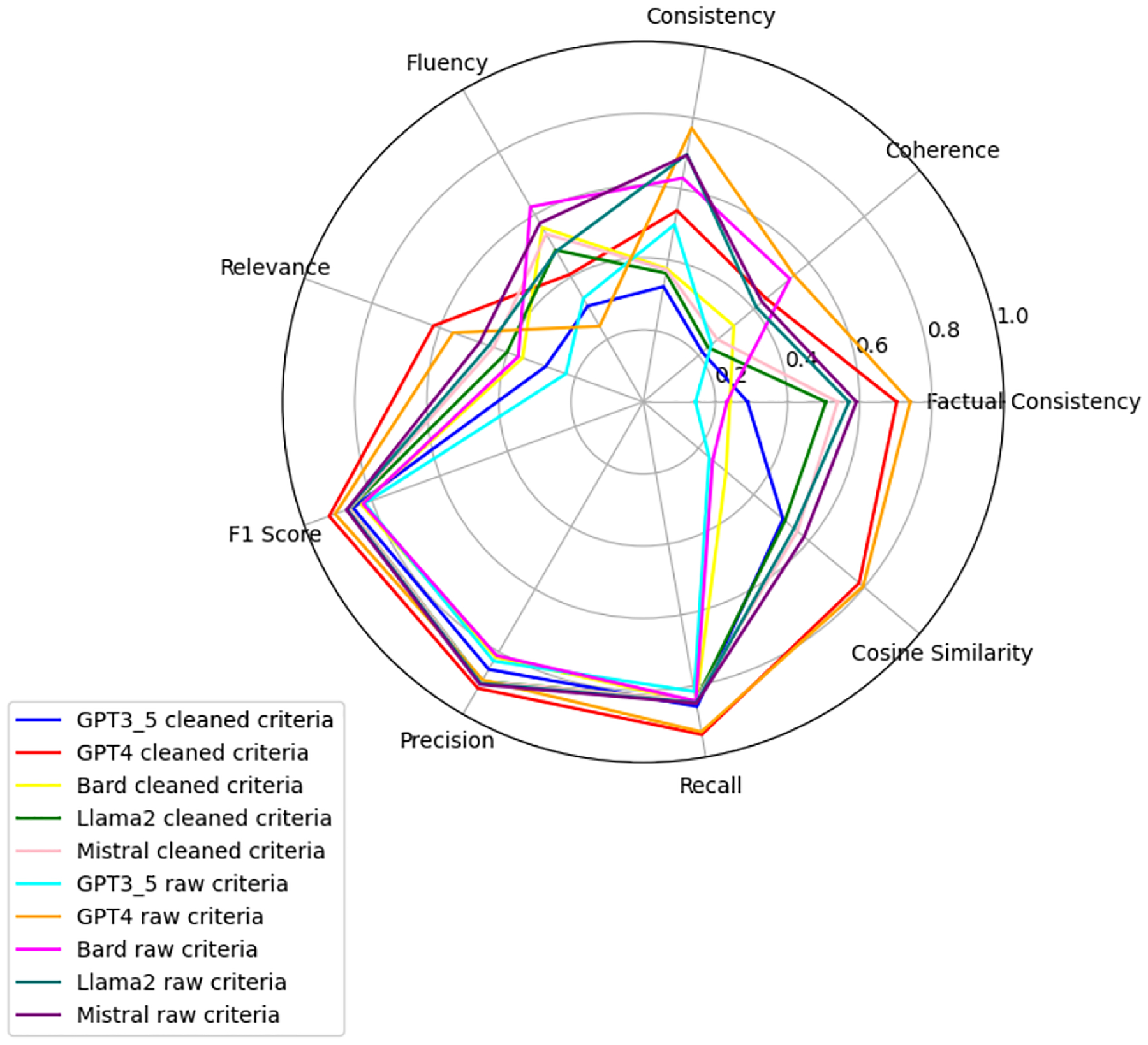
Platelets: all models.

**Figure 11.**
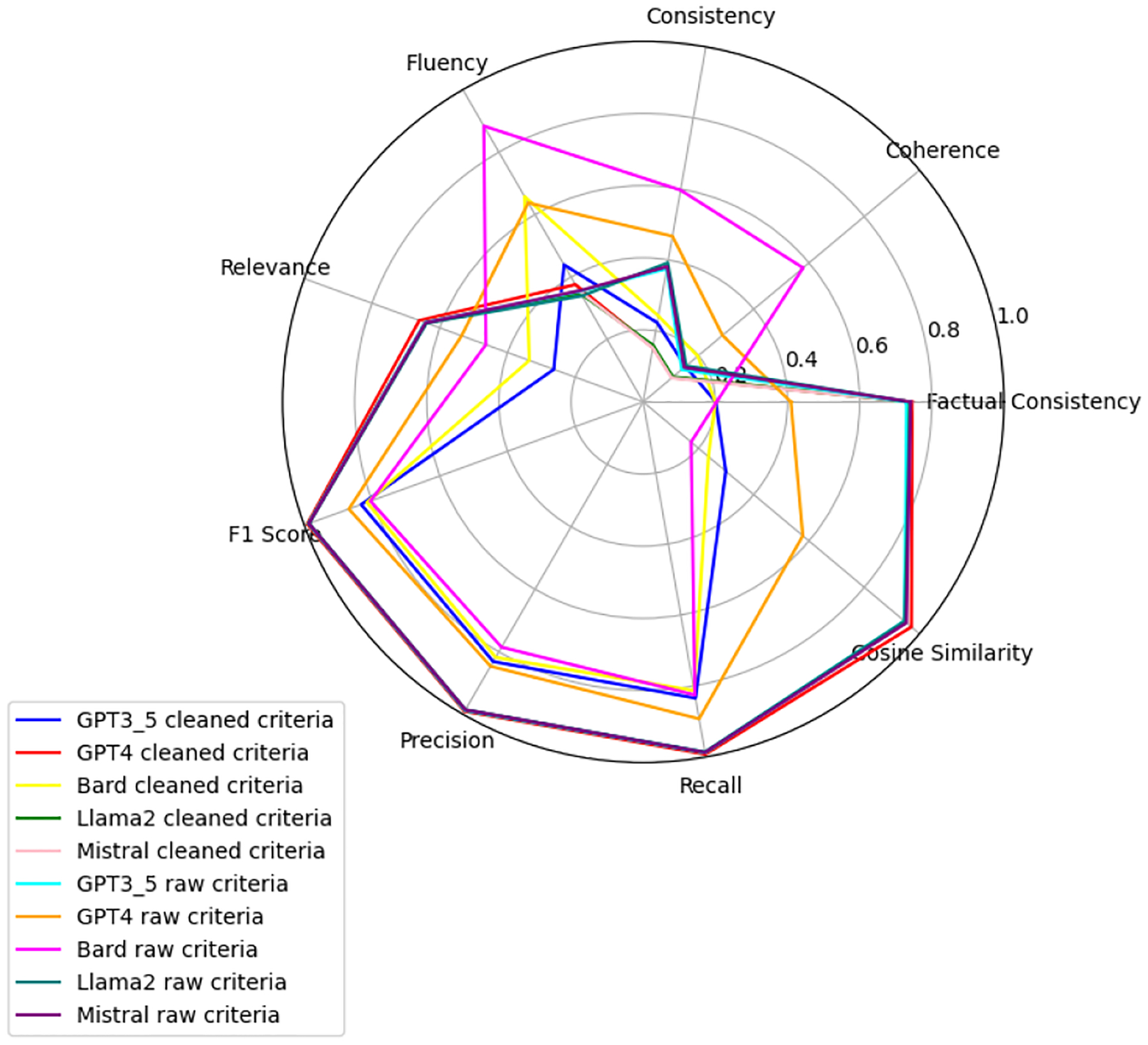
WBC: all models.

We observe that the evaluation of large language models across six health-related categories—Age, BMI, Disease, Gender, Platelets, and WBC—reveals significant insights into the models’ performances with raw and clean datasets. This comprehensive evaluation employs the TOPSIS method alongside human validation scores, underscoring the varying impact of data quality on model accuracy and reliability. Each category—Age, BMI, Disease, Gender, WBC, and Platelets—underscores different facets of model performance, with detailed evaluations presented in Appendix A. Figures 6–11 present radar plots comparing the performance of four large language models (GPT-3.5, Bard, Llama2, and Mistral) on tasks related to Age, BMI, Disease, Gender, Platelets, and WBC, under both “cleaned” and “raw” criteria. Each plot highlights variations in metrics such as consistency, coherence, factual consistency, cosine similarity, recall, precision, and F1 score, illustrating how certain models excel in specific dimensions while others perform better in different areas.

### 3.1. Evaluation of Disease Extraction

For disease extraction validation, we established a predefined set of target diseases as our ground truth: HIV, hepatitis, cancer, diabetes, and cardiovascular disease. When evaluating large language models (LLMs) on this task, we observed considerable variation in their outputs. Models responded in several different formats: some provided narrative descriptions (e.g., “The text mentions hepatitis, HIV, and diabetes as exclusion criteria”), others returned structured lists (e.g., “[hepatitis, HIV, and diabetes]”), and certain models simply repeated the input prompt or incorrectly indicated that no diseases were mentioned in the text. To standardize our evaluation, we defined the optimal (cleaned) answer for the example case as [hepatitis, HIV, and diabetes], allowing for consistent comparison across different model outputs regardless of their formatting inconsistencies. This standardization was essential for quantitative assessment of extraction accuracy across various LLMs and prompt strategies.

Table 1 summarizes the evaluation metrics used to assess the quality of disease extraction.

### 3.2. Comparison of Language Models

GPT4 emerges as a resourceful model, demonstrating superior capabilities in processing criteria related to Age, Gender, and Platelets with both raw and clean datasets. For Age and Gender, GPT4’s performance with raw data ranks first, indicating its advanced linguistic analysis capabilities and a nuanced understanding of diverse expressions in patient data. Its standout performance in Platelets count assessment with clean data emphasizes the importance of data quality in achieving accurate health assessments, especially in predicting clotting disorders or bleeding conditions. This balance between processing capabilities with raw data and the enhancement provided by clean datasets is a recurrent theme, notably improving the model’s performance in disease pattern identification as well.

In contrast, Bard exhibits a slight edge over GPT4 in the BMI category with raw data, highlighting its robustness in handling complex nutritional indicators. This suggests Bard’s potential utility in environments with limited preprocessing capabilities. However, a comprehensive analysis incorporating Platelets criteria reveals that GPT4’s clean data processing in this area achieves a high human validation score, contrasting with other models and underlining the critical role of clean data for accurate health assessments.

The evaluation further highlights discrepancies between algorithmic efficiency and human judgment, particularly in the WBC count assessment where Bard leads in the TOPSIS ranking for raw data processing, yet llama_2 is preferred according to human validation scores. This indicates llama_2’s nuanced understanding of subtle patterns in WBC data, aligning more closely with expert judgments—crucial for diagnosing infections or blood disorders.

Detailed evaluations for these categories are presented in Tables A1–A6 in Appendix A. Table 2, We explained our results in detail.

**Age Criteria**: GPT4 stands out, with its performance on raw data receiving a high MCDA score and an almost perfect human validation score of 0.93. When clean data are used, GPT4 achieves a human validation score of 1.00, indicating flawless accuracy.

**BMI Criteria**: Bard’s analysis of raw data is top-ranked by the MCDA method with a human validation score of 0.80, showcasing its robustness in interpreting complex nutritional health indicators. GPT4 with clean data achieves a slightly higher human validation score of 0.84.

**Disease Criteria**: GPT4’s raw data approach initially ranks highest by MCDA, with a human validation score of 0.51, improving to 0.56 with clean data, emphasizing the impact of data quality.

**Gender Criteria**: GPT4’s raw data processing ranks highest by MCDA for gender identification, with a human validation score of 0.59, matched by GPT3.5 Raw and GPT4 Clean, showcasing superior linguistic analysis capabilities.

**WBC Criteria**: Bard’s raw data processing leads in MCDA ranking but receives a notably low human validation score of 0.05, suggesting areas for model improvement. Conversely, GPT4 with clean data secures a near-perfect human validation score of 0.99, indicating its precision in WBC count assessment.

**Platelets Criteria**: GPT4’s processing of clean data excels in both MCDA and human validation scores, achieving 0.92, highlighting the significance of data quality for accurate assessments.

This comprehensive analysis elucidates the strengths and weaknesses of different models across multiple health-related criteria, revealing how some models excel with raw data while others achieve remarkable accuracy with clean datasets, as reflected in their human validation scores. These insights serve as a crucial reference for understanding model performance in health data analysis, emphasizing the importance of human validation in verifying and contextualizing algorithmic assessments. This comprehensive evaluation not only illustrates the nuanced capabilities of large language models in healthcare data analysis but also emphasizes the critical role of data quality. It highlights the necessity for balanced consideration between algorithmic predictions and human evaluation in healthcare applications. The insights drawn from this analysis underscore the importance of model selection tailored to specific healthcare tasks, guided by both data quality and the intrinsic capabilities of each model. Future research directions should focus on enhancing models’ data processing capabilities, exploring automated preprocessing techniques, and broadening the scope of evaluation to include more diverse datasets and health conditions, aiming to maximize the reliability and applicability of these models in real-world healthcare settings.

## 4. Discussion

The present study aimed to evaluate the performance of LLMs in extracting patient characteristics from free-text clinical records, addressing a critical gap in the existing literature [51]. Accurate and efficient extraction of patient characteristics is crucial for various healthcare applications, including clinical trial recruitment, personalized medicine, and epidemiological research [52]. Our comprehensive evaluation framework, which assessed LLM performance across various dimensions, including semantic textual similarity, factual consistency, coherence, prevalence, fluency, and consistency, provides valuable insights into the strengths and limitations of different models and their potential to advance the field of patient characteristics extraction.

One of the key contributions of our work is the focus on a diverse set of patient characteristics, including age with 336 patients ranging from 0 to 90 years old, BMI with 43 patients among 3 categories, disease with 280 patients, gender with 341 patients, WBC count with 37 patients, and platelets count with 61 patients. Previous studies have often focused on a limited set of characteristics or have not provided detailed evaluations across multiple dimensions [53]. By assessing LLM performance on this diverse set of characteristics, we provide a more comprehensive understanding of their capabilities and limitations in extracting clinically relevant information from free-text records. This is particularly important given the heterogeneity of clinical data and the need for models that can handle a wide range of patient characteristics [52].

Our results demonstrate that GPT4, particularly when feed on clean datasets, exhibits superior performance in accurately extracting patient characteristics compared to other models like GPT3.5, llama2, and Bard. This finding builds upon previous work that has highlighted the potential of LLMs for clinical information extraction [54] and extends it by providing a more nuanced evaluation of model performance across different characteristics and dataset conditions. The strong performance of GPT4 suggests that it could be a valuable tool for automating patient characteristics extraction in clinical settings, potentially saving time and resources compared to manual chart review [55].

The use of large language models (LLMs) for data annotation in healthcare raises critical privacy and ethical considerations. One primary concern is the handling of sensitive patient data. While LLMs offer significant efficiency improvements in data extraction, they must comply with regulatory frameworks such as HIPAA and GDPR to ensure that patient confidentiality is maintained. Moreover, there is a risk of model-generated biases, as LLMs may reflect and propagate existing disparities present in training data, potentially leading to inaccurate or inequitable annotations. Ethical deployment of LLMs in healthcare necessitates ongoing human oversight to validate outputs, mitigate biases, and ensure transparency in decision-making processes. Furthermore, researchers and practitioners must implement stringent data anonymization techniques and secure processing environments to prevent unauthorized access or misuse of annotated clinical data. Addressing these ethical challenges is crucial to fostering trust and ensuring that LLM-driven data annotation aligns with principles of fairness, accountability, and patient-centric care [56].

However, our study also highlights the challenges and considerations involved in applying LLMs to patient characteristics extraction. We found that data quality, as reflected in the use of clean versus raw datasets, had a significant impact on model performance, particularly for complex characteristics like disease and platelets count. This echoes previous findings on the importance of data preprocessing and quality control in clinical natural language processing [57] and underscores the need for careful data curation when applying LLMs to patient characteristics extraction. Our work contributes to the growing body of research on best practices for data preparation in clinical AI applications [58].

Another important contribution of our study is the incorporation of human validation alongside algorithmic evaluation. While previous studies have often relied solely on automated metrics like F1-scores or accuracy [59], we found that human validation provided additional insights and sometimes diverged from the algorithmic rankings. This highlights the importance of involving domain experts in the evaluation process and not relying solely on automated metrics to assess model performance [60]. Our findings suggest that a hybrid approach, combining algorithmic efficiency with human judgment, may be the most effective strategy for ensuring the accuracy and reliability of patient characteristics extraction in clinical settings.

In terms of clinical implications, our work demonstrates the potential of LLMs to automate and streamline the process of the extraction of patient characteristics from free-text records. This automation can lead to significant benefits in various healthcare applications. For instance, in clinical trial recruitment, LLMs can rapidly analyze electronic health records (EHRs) to identify eligible patients based on specific inclusion and exclusion criteria, significantly reducing the time and resources required for manual screening [61]. Furthermore, LLMs can enhance precision medicine by providing clinicians with a more comprehensive view of a patient’s medical history and risk factors. By synthesizing information from diverse sources such as clinical notes, lab results, and imaging reports, LLMs enable the creation of personalized treatment plans tailored to individual patient profiles [62].

Additionally, LLMs can support clinical decision-making by offering real-time insights and recommendations based on the latest medical research and patient data [63]. In practice, this means that clinicians can receive evidence-based suggestions for diagnosis and treatment options during patient consultations, enhancing the quality of care. Moreover, LLMs can facilitate the management of chronic diseases by continuously monitoring patient data and predicting potential complications before they arise [64]. This proactive approach allows for timely interventions, improving long-term health outcomes for patients with conditions such as diabetes and hypertension. In real-world settings, hospitals have implemented LLM-driven systems that alert healthcare providers to changes in patient conditions, enabling swift and informed decision-making.

However, our study also highlights the need for rigorous evaluation and validation of LLMs before deploying them in clinical settings to ensure their safety and effectiveness. Ensuring that these models are free from biases and capable of handling sensitive patient information responsibly is paramount for their successful integration into healthcare systems.

One of the main limitations of our study is its focus on a specific set of patient characteristics and clinical settings, which may restrict the generalizability of our findings to other healthcare domains and diverse patient populations. Future research should explore the applicability of our evaluation framework across a broader range of healthcare settings and patient demographics to enhance the robustness and external validity of the results.

Additionally, our benchmarking study was constrained by the context window size of open source models, limiting the input length to a maximum of 4096 tokens. This constraint contrasts sharply with the capabilities of models like GPT4 and GPT3.5, which support much larger inputs. The limited context window may affect the models’ ability to accurately extract patient characteristics from lengthy clinical texts, potentially underestimating their true performance in real-world scenarios.

Furthermore, we encountered challenges related to content and bias guardrail mechanisms, particularly with Bard. These mechanisms, designed to mitigate biases, prevented us from feeding certain prompts to the models. For instance, Bard exhibited restrictions on inputs related to gender and age. While these guardrails aim to reduce bias and ensure ethical usage, they significantly limited the scope of prompts we could test, highlighting a trade-off between ethical considerations and the breadth of conversational topics available for benchmarking [65].

Moreover, future work should aim to analyze the underlying reasons why certain models, such as GPT4, outperform others in specific criteria, and why models like Bard excel in particular categories such as BMI. This analysis would provide a richer understanding of the models’ behaviors and their context-dependent performance in knowledge extraction tasks. Investigating these performance discrepancies could inform the development of more sophisticated models tailored to specific healthcare applications. Finally, expanding our study to include more diverse clinical datasets would enhance the generalizability of our results and provide a more comprehensive evaluation of LLM capabilities across various healthcare settings. Incorporating datasets from different medical disciplines, geographic regions, and patient populations would ensure that the evaluation framework remains relevant and effective in diverse real-world applications.

## 5. Conclusions

In conclusion, our study makes important contributions to the field of patient characteristics extraction by providing a comprehensive evaluation of LLM performance across a diverse set of characteristics and dataset conditions. We demonstrate the potential of models like GPT4 to automate and streamline this process, while also highlighting the challenges and considerations involved in their deployment. By proposing a hybrid evaluation approach that combines algorithmic efficiency with human judgment, we provide a framework for ensuring the accuracy and reliability of LLMs in clinical settings. Ultimately, our work lays the foundation for future research and development in this area, with the goal of leveraging LLMs to improve patient care and advance precision medicine.

## Figures and Tables

**Table 1.** Evaluation metrics for disease extraction.

Metric	Score
Factual Consistency	0.0369
Coherence	0.8462
Consistency	0.9058
Fluency	0.9475
Relevance	0.4792
Overall	0.7947
F1-Score	0.9185
Precision	0.9163
Recall	0.9207
Cosine Similarity	0.8947
Accuracy	0.8000

**Table 2.** Comparison of model performance across various health-related criteria. Models are evaluated through the TOPSIS method and human validation scores for processing datasets related to Age, BMI, Disease, Gender, WBC, and Platelets.

Criteria	Top Rank Measure	Model	Human Validation Score
Age	MCDA	GPT4 Raw	0.93
Age	Human Validation	GPT4 Clean	1.00
BMI	MCDA	Bard Raw	0.80
BMI	Human Validation	GPT4 Clean	0.84
Disease	MCDA	GPT4 Raw	0.51
Disease	Human Validation	GPT4 Clean	0.56
Gender	MCDA	GPT4 Raw	0.59
Gender	Human Validation	GPT3.5 Raw	0.59
Gender	Human Validation	GPT4 Clean	0.59
WBC	MCDA	Bard Raw	0.05 *
WBC	Human Validation	GPT4 Clean	0.99
Platelets	MCDA	GPT4 Clean	0.92
Platelets	Human Validation	GPT4 Clean	0.92

* Low validation score for Bard Raw in WBC category indicates potential model improvement or data preprocessing areas.

## Data Availability

The original contributions presented in this study are included in the article. Further inquiries can be directed to the corresponding author.

## Appendix A

In evaluating language models, various metrics are employed to measure their performance and alignment with human expectations. Below is a list of abbreviations for these metrics:

- **HV**: Human Validation—assesses how well the model’s outputs align with human judgment.
- **F1**: F1-Score—measures the balance between precision and recall, indicating the model’s accuracy [66].
- **Prec.**: Precision—gauges the correctness of the model’s positive predictions [67].
- **Cos. Sim.**: Semantic Textual Similarity—calculates the similarity between two vectorized texts [68].
- **Fact. Cons.**: Factual Consistency—verifies the accuracy of the information provided by the model [69].
- **Coh.**: Coherence—evaluates the logical and smooth flow of text [70].
- **Cons.**: Consistency—checks for uniform responses to similar inputs [71].
- **Flu.**: Fluency—assesses the smoothness and naturalness of the text [72].
- **Relev.**: Relevance—measures how well the model’s outputs correspond to the input prompts [73].

**Table A1.** Evaluation metrics for different models for gender criteria.

Model	Rank	HV	F1	Prec.	Recall	Cos. Sim.	Fact. Cons.	Coh.	Cons.	Flu.	Relev.
GPT4 Raw *	1	0.59	0.95	0.95	0.95	0.87	0.70	0.41	0.63	0.43	0.41
GPT3_5 Raw	2	0.51	0.93	0.93	0.94	0.69	0.54	0.37	0.55	0.50	0.32
Llama_2 Raw	3	0.49	0.94	0.94	0.95	0.71	0.54	0.35	0.57	0.41	0.33
Mistral Raw	4	0.42	0.93	0.92	0.93	0.57	0.41	0.30	0.52	0.40	0.27
GPT4 Clean *	5	0.59	0.95	0.95	0.95	0.79	0.62	0.14	0.21	0.46	0.35
Llama_2 Clean	6	0.51	0.95	0.95	0.95	0.76	0.59	0.14	0.21	0.41	0.36
GPT3_5 Clean	7	0.54	0.94	0.93	0.95	0.71	0.55	0.14	0.21	0.49	0.32
Mistral Clean	8	0.43	0.93	0.92	0.94	0.61	0.45	0.12	0.18	0.40	0.28
Bard Raw	9	0.07	0.86	0.85	0.87	0.31	0.25	0.38	0.56	0.39	0.19
Bard Clean	10	0.08	0.86	0.84	0.87	0.28	0.23	0.21	0.28	0.38	0.18

* Based on the above table, it is noted that the GPT4 model for raw data is the top-ranked model for the gender criteria based on the MCDA metrics, but the GPT4 model for clean data is ranked highest based on human validation score, which is 0.59.

**Table A2.** Evaluation metrics for different models for age criteria.

Model	Rank	HV	F1	Prec.	Recall	Cos. Sim.	Fact. Cons.	Coh.	Cons.	Flu.	Relev.
GPT4 Raw *	1	0.93	0.97	0.97	0.97	0.92	0.80	0.78	0.87	0.39	0.75
GPT3_5 Raw	2	0.80	0.96	0.96	0.95	0.85	0.72	0.71	0.82	0.36	0.69
GPT4 Clean	3	1.00	0.97	0.97	0.97	0.92	0.80	0.37	0.47	0.39	0.76
GPT3_5 Clean	4	0.86	0.96	0.96	0.96	0.88	0.76	0.35	0.45	0.37	0.72
Bard Raw	5	0.77	0.84	0.83	0.85	0.49	0.37	0.40	0.53	0.29	0.31
Mistral Raw	6	0.16	0.87	0.88	0.86	0.40	0.24	0.28	0.49	0.34	0.30
Bard Clean	7	0.08	0.85	0.85	0.85	0.46	0.30	0.25	0.33	0.38	0.31
Llama_2 Raw	8	0.11	0.89	0.89	0.89	0.57	0.28	0.33	0.45	0.18	0.27
Llama_2 Clean	9	0.38	0.88	0.89	0.88	0.55	0.25	0.19	0.25	0.18	0.25
Mistral Clean	10	0.12	0.86	0.87	0.85	0.36	0.19	0.15	0.21	0.32	0.26

* Based on the above table, it is noted that the GPT4 model for raw data is the top-ranked model for the age criteria based on the MCDA metrics and has the highest human validation score of 0.93.

**Table A3.** Evaluation metrics for different models for BMI criteria.

Model	Rank	HV	F1	Prec.	Recall	Cos. Sim.	Fact. Cons.	Coh.	Cons.	Flu.	Relev.
Bard Raw *	1	0.80	0.93	0.92	0.94	0.76	0.55	0.65	0.77	0.51	0.60
GPT4 Raw	2	0.81	0.91	0.89	0.92	0.68	0.59	0.62	0.78	0.34	0.59
GPT4 Clean *	3	0.84	0.93	0.93	0.94	0.76	0.62	0.45	0.52	0.32	0.66
Bard Clean	4	0.77	0.93	0.92	0.93	0.75	0.62	0.39	0.46	0.52	0.64
GPT3_5 Clean	5	0.74	0.90	0.89	0.91	0.63	0.58	0.42	0.48	0.41	0.62
GPT3_5 Raw	6	0.47	0.87	0.87	0.87	0.47	0.35	0.48	0.67	0.43	0.47
Mistral Raw	7	0.07	0.84	0.87	0.81	0.26	0.15	0.23	0.46	0.35	0.24
Llama_2 Raw	8	0.07	0.85	0.87	0.83	0.47	0.17	0.20	0.35	0.29	0.19
Llama_2 Clean	9	0.16	0.85	0.88	0.83	0.43	0.22	0.18	0.25	0.29	0.23
Mistral Clean	10	0.09	0.83	0.85	0.81	0.23	0.16	0.13	0.20	0.36	0.27

* Based on the above table, it is noted that the Bard model for raw data is the top-ranked model for the BMI criteria based on the MCDA metrics, but the GPT4 model for clean data is ranked highest based on human validation score, which is 0.84.

**Table A4.** Evaluation metrics for different models for disease criteria.

Model	Rank	HV	F1	Prec.	Recall	Cos. Sim.	Fact. Cons.	Coh.	Cons.	Flu.	Relev.
GPT4 Raw *	1	0.51	0.93	0.93	0.93	0.86	0.48	0.58	0.73	0.87	0.63
GPT4 Clean *	2	0.56	0.94	0.94	0.94	0.88	0.53	0.44	0.55	0.87	0.67
GPT3_5 Clean	3	0.47	0.92	0.93	0.91	0.79	0.47	0.39	0.50	0.79	0.59
GPT3_5 Raw	4	0.38	0.91	0.92	0.91	0.76	0.41	0.52	0.69	0.78	0.54
Llama_2 Raw	5	0.02	0.86	0.85	0.88	0.58	0.13	0.43	0.59	0.80	0.38
Mistral Raw	6	0.03	0.87	0.85	0.89	0.61	0.15	0.39	0.55	0.83	0.38
Mistral Clean	7	0.02	0.88	0.86	0.91	0.66	0.16	0.35	0.42	0.89	0.40
Llama_2 Clean	8	0.03	0.88	0.86	0.90	0.62	0.13	0.36	0.44	0.85	0.41
Bard Raw	9	0.02	0.83	0.85	0.81	0.40	0.24	0.26	0.44	0.27	0.30
Bard Clean	10	0.04	0.82	0.84	0.81	0.41	0.25	0.20	0.25	0.30	0.31

* Based on the above table, it is noted that the GPT4 model with raw data is the top-ranked model for the disease criteria based on the MCDA metrics and the GPT4 model with clean data has the highest human validation score of 0.56.

**Table A5.** Evaluation metrics for different models for platelets criteria.

Model	Rank	HV	F1	Prec.	Recall	Cos. Sim.	Fact. Cons.	Coh.	Cons.	Flu.	Relev.
GPT4 Clean *	1	0.92	0.93	0.92	0.94	0.78	0.70	0.45	0.54	0.41	0.62
GPT4 Raw	2	0.82	0.91	0.89	0.93	0.80	0.74	0.55	0.77	0.24	0.56
Mistral Raw	3	0.52	0.88	0.90	0.85	0.58	0.59	0.43	0.69	0.57	0.48
Llama_2 Raw	4	0.44	0.87	0.90	0.85	0.55	0.57	0.41	0.70	0.48	0.46
Mistral Clean	5	0.44	0.87	0.90	0.84	0.56	0.54	0.27	0.37	0.54	0.44
Llama_2 Clean	6	0.36	0.87	0.89	0.84	0.51	0.51	0.23	0.36	0.49	0.40
Bard Raw	7	0.23	0.83	0.81	0.84	0.25	0.23	0.53	0.63	0.62	0.37
Bard Clean	8	0.16	0.83	0.82	0.85	0.30	0.24	0.33	0.38	0.56	0.36
GPT3_5 Clean	9	0.21	0.86	0.86	0.86	0.51	0.29	0.21	0.32	0.31	0.29
GPT3_5 Raw	10	0.10	0.82	0.83	0.82	0.24	0.14	0.25	0.50	0.33	0.23

* Based on the provided table, it is noted that the GPT4 Clean model is the top-ranked model for the platelets criteria, while also having the highest human validation score of 0.92.

**Table A6.** Evaluation metrics for different models for WBC criteria.

Model	Rank	HV	F1	Prec.	Recall	Cos. Sim.	Fact. Cons.	Coh.	Cons.	Flu.	Relev.
Bard Raw	1	0.05	0.80	0.79	0.82	0.17	0.20	0.58	0.60	0.88	0.46
GPT4 Raw	2	0.76	0.87	0.85	0.89	0.58	0.41	0.29	0.47	0.64	0.54
Llama_2 Raw *	3	0.94	0.99	0.99	0.99	0.95	0.74	0.16	0.39	0.34	0.64
Mistral Raw	4	0.95	0.99	0.99	0.99	0.95	0.74	0.15	0.38	0.36	0.64
GPT3_5 Raw	5	0.93	0.99	0.99	0.99	0.95	0.73	0.14	0.38	0.35	0.64
GPT4 Clean	6	0.99	0.99	0.99	0.99	0.97	0.75	0.11	0.15	0.38	0.66
Llama_2 Clean	7	0.95	0.99	0.99	0.99	0.95	0.74	0.11	0.16	0.35	0.64
Mistral Clean	8	0.94	0.99	0.99	0.99	0.95	0.74	0.10	0.15	0.36	0.64
Bard Clean	9	0.03	0.81	0.82	0.81	0.24	0.20	0.20	0.24	0.66	0.34
GPT3_5 Clean	10	0.16	0.83	0.83	0.83	0.30	0.20	0.15	0.22	0.44	0.26

* Based on the above table, it is noted that the GPT4 model with clean data is the top-ranked model for the WBC (white blood cell) criteria, while the GPT4 model with raw data has the highest human validation score of 0.84.
